# Influence of type of violence on women’s help-seeking behaviour: Evidence from 10 countries in sub-Saharan Africa

**DOI:** 10.1371/journal.pone.0297308

**Published:** 2024-03-08

**Authors:** Castro Ayebeng, Kwamena Sekyi Dickson, Edward Kwabena Ameyaw, Kenneth Setorwu Adde, Jones Arkoh Paintsil, Sanni Yaya

**Affiliations:** 1 Department of Population and Health, University of Cape Coast, Cape Coast, Ghana; 2 Institute of Policy Studies and School of Graduate Studies, Lingnan University, Tuen Mun, Hong Kong; 3 L & E Research Consult Ltd, Upper West Region, Ghana; 4 Department of Economics, Howard University, Washington, DC, United States of America; 5 School of International Development and Global Studies, University of Ottawa, Ottawa, Ontario, Canada; 6 The George Institute for Global Health, Imperial College London, London, United Kingdom; Wits University: University of the Witwatersrand Johannesburg, SOUTH AFRICA

## Abstract

**Background:**

Intimate partner violence (IPV) is a major public health concern that mostly impacts women’s health and social well-being. This study explored how the various types of IPV (physical, sexual, and emotional) including women’s experience of childhood violence influence their help-seeking behavior in sub-Saharan Africa (SSA).

**Methods:**

We analyzed data from the most recent Demographic and Health Surveys (DHS), carried out between 2018 and 2021. The outcome variable was help-seeking behavior. Descriptive and inferential analyses were carried out. The descriptive analysis looked at the bivariate analysis between the country and outcome variables. Using a binary logistic regression model, a multivariate analysis was utilized to determine the association between the outcome variable and the explanatory variables. Binary logistic regression modelling was used based on the dichotomous nature of the outcome variable. The results were sample-weighted to account for any under- or over-sampling in the sample.

**Results:**

The proportion of women who sought help for intimate partner violence was 36.1 percent. This ranged from 19.2 percent in Mali to 49.6 percent in Rwanda. Women who experienced violence in childhood (OR = 0.75, CI = 0.69, 0.82) have a lower likelihood of seeking help compared to those who did not experience violence in their childhood. Women who had experienced emotional violence (OR = 1.94, CI = 1.80, 2.08), and physical violence (OR = 1.37, CI = 1.26, 1.48) have a higher likelihood of seeking help compared to those who have not. Women with secondary educational levels (aOR = 1.13, CI = = 1.02, 1.24) have a higher likelihood of seeking help compared to those with no education. Cohabiting women have a higher likelihood (aOR = 1.22, CI = 1.10, 1.35) of seeking help compared to married women.

**Conclusion:**

The study highlights the importance of early identification of IPV and fit-for-purpose interventions to demystify IPV normalization to enhance women’s willingness to seek help. The study’s findings suggest that education is crucial for increasing women’s awareness of the legalities surrounding IPV and available structures and institutions for seeking help.

## Background

Intimate partner violence (IPV) is a major public health concern that mostly impacts women’s health and social well-being, and, largely, the violation of their fundamental human rights [1]. It transcends regional, socio-economic, and cultural boundaries. Intimate partner violence encompasses acts of physical, sexual, controlling behaviours, and emotional (psychological) abuse that are perpetrated by an intimate partner [2]. IPV is a complex phenomenon that can have severe consequences for the victims and the perpetrator, making it imperative to understand the different types of violence and their impact on help-seeking behaviour.

Globally, since 1979 to date, there have been several commitments both at the international and local levels to mitigate all forms of violence against women and its associated effects. Notable among these international commitments include the ratification of the Convention on the Elimination of all forms of Discrimination Against Women (CEDAW) [3], the Beijing Platform for Action (BPFA) which serves as a milestone document for advancing women’s rights and gender equality [4], and the adoption of the 17 Sustainable Development Goals (SDGs) in the year 2015 of which target 5.2 envision to eliminate all forms of violence against all women and girls in the public and private spheres by 2030 [5]. Specifically, the first indicator of this target (5.2.1) primarily focuses on intimate partner violence, requiring countries to regularly report on "the proportion of ever-partnered women and girls aged 15 years and older subjected to physical, sexual, or psychological violence by a current or former male intimate partner [5].

Notwithstanding these concerted efforts, global estimates indicate that about 641 million, thus, 1 in 3 (27%) women and girls aged 15–49 years have encountered some form of physical and/or sexual violence by an intimate partner during their lifetime [1]. The prevalence of IPV is unequally distributed across the globe, being more pronounced in low and middle-income countries. In 2018, the Global Burden of Disease estimated the regional prevalence of lifetime and past year physical and/or sexual IPV among ever-married or partnered women aged 15–49 years. The study highlights that the highest prevalence of IPV was in the regions of central sub-Saharan Africa (32%) and Oceania (29%), followed by eastern sub-Saharan Africa (24%) and south Asia (19%) [6].

Intimate partner violence is associated with dire consequences on the physical and mental health of victims, encompassing injuries, depression, anxiety, unwanted pregnancies, and sexually transmitted infections, posttraumatic stress disorder, chronic mental illness among others, and the extreme of leading to suicidality or death [7–10]. In 2018, a global estimate indicates that 38–50% of women who are being murdered are committed by intimate partners [6]. Beyond the immediate consequences of IPV, it also contributes significantly to social and economic costs for governments, communities, and individuals [11].

Among the various forms of IPV, physical violence is the most noticeable form, involving physical acts such as hitting, pushing, choking, and or slapping [2]. This often results in observable injuries, making it easier for victims to seek help. Studies have revealed that victims of physical violence are more likely to seek help from sources such as friends, family, and healthcare providers compared to victims of other forms of violence [12, 13]. Sexual violence is another form of IPV that involves unwanted sexual contact, coercion, or abuse. it is evident from research that victims of sexual violence are less likely to seek help compared to victims of physical violence [14]. Victims of IPV may be reluctant to seek help because of the fear that they will not be believed for fear of perpetual violence, fear of stigmatization, and concern about children [13, 15]. Furthermore, emotional, or psychological abuse of IPV can have long-lasting effects on victims. It includes behaviours such as controlling, intimidating, isolating, stalking, threatening, or humiliating the victim [1].

Another critical dimension of IPV that has received less attention is spiritual abuse. It is a form of abuse that involves using religious or spiritual beliefs to manipulate, control, or harm a partner. Perpetrators may employ tactics such as using religious doctrines to justify abusive behavior, restricting a partner’s religious practices, or using spiritual beliefs to demean and manipulate the victim [16]. Also, financial or economic abuse highlights the occurrence of IPV when one partner controls the other’s financial resources, limits their access to money, or uses financial means to exert power and control [17]. Tactics may include withholding money, preventing access to bank accounts, interfering with employment or education opportunities, or coercing a partner into financial dependence. Recognizing the full spectrum of abuse, including spiritual and financial aspects, is crucial for comprehensive understanding and effective intervention in cases of IPV.

Despite the avalanches of empirical evidence on IPV, the scope of most studies examining the link between IPV and help-seeking behaviour is limited to country-specifics, thereby limiting our understanding of how the phenomenon varies across countries in SSA [13]. Also, extant literature on IPV and help-seeking behaviour is partly limited to one or two forms of IPV [15, 18]. Hence this study attempts to fill these gaps in knowledge by using nationally representative cross-sectional data of ten SSA countries with the most current (2018–2021) Demographic and Health Survey (DHS) data to explore holistically how the various types of IPV (physical, sexual, and emotional) including women’s experience of childhood violence influence their help-seeking behaviour. In addition, this study further considers the interplay between women’s socio-demographic characteristics (such as age, residence, education, wealth status, marital status, and occupation) and sociocultural factors (justification for wife beaten, and partner domineering attitude) with their likelihood to seek help for intimate partner violence.

The influence of different forms of violence on help-seeking behaviour is a crucial aspect of IPV. Hence, exploring the nuances among violence types would present more precise evidence about intimate partner violence and the mechanisms that link it to the help-seeking decisions of victims. Also, this information could help in developing effective interventions and support systems to assist victims in escaping abusive relationships and seeking help.

## Methods

### Data source

The study analysed data from the most recent Demographic and Health Surveys (DHS), carried out between 2018 and 2021 in Sub-Saharan Africa (SSA). Countries were selected based on data availability for the variables of interest. The DHS data comprises cross-sectional and nationally representative information obtained through probability multi-stage samples of households in low- and middle-income countries (LMICs) [19]. In the chosen households, the survey selected samples of women of reproductive age (15 to 49 years) for interviews. The DHS is ideal for our study because it collects comprehensive information on various topics, including fertility, intimate partner violence, family planning, infant and child mortality, maternal health care (antenatal care, delivery, and postnatal care), and child care (nutrition, abuse). Women who have endured any type of IPV were included in the study. The surveys employed consistent methodologies for data collection and utilized similar questionnaires, facilitating cross-country comparisons [20]. A total of 174,280 women were interviewed. For this study, only women who have experienced intimate partner violence were included in the analyses. All women who have never married or in any sexual union were not included in the sample. A final sample size of 18,617 women was drawn from 10 different countries. The MEASURE DHS approved the use of the data set after reviewing our concept note.

### Study variables and measurements

#### Outcome variable

The outcome variable for this study was “help-seeking after experiencing IPV.” This variable was derived from the question, “Did the respondent seek help from anyone about the IPV?” The response was captured as “no” and “yes.”

#### Explanatory variables

Following theoretical and empirical literature, twelve explanatory variables were used [7, 13, 21]. The main explanatory variables include experienced childhood violence, emotional violence, physical violence, and sexual violence. The experienced childhood violence was created in response to questions “ever physically hurt by father” and “ever physically hurt by mother” (no, yes). The experience of any emotional violence by husband/partner, the experience of any physical violence by husband/partner, and the experience of any sexual violence by husband/partner was measured as yes and no. Justification of wife-beating was a composite variable derived from reasons due to 1. neglect of a child; 2. burning of food; 3. arguing with husband/partner; 4. refusal to have sex with husband/partner; and 5. going out without permission.

These were measured as yes = 1 or no = 0. An index was created with all the “yes” and “no” answers, with scores ranging from 0 to 5. The 0 scores were labelled as “no,” and 1 to 5 were labelled as “yes.” The Cronbach’s alpha was 0.85. Partner domineering attitude was a composite variable derived from 1. Partner jealous if respondent talks with other men; 2. Partner accuses respondent of unfaithfulness; 3. Partner does not permit respondents to meet female friends; 4. Partner tries to limit respondent’s contact with family; 5. Partner insists on knowing where the respondent is. These were measured as yes = 1 or no = 0. An index was created with all the “yes” and “no” answers, with scores ranging from 0 to 5. The 0 scores were labelled as “no,” and 1 to 5 were labelled as “yes.” The Cronbach’s alpha was 0.72. Also, sociodemographic variables such as women’s age (15–19,20–24,25–29,30–34,35–39,40–44,45–49), occupation (working, not working), level of education (no education, primary, secondary, higher) wealth status (poorest, poorer, middle, richer, richest), residence (rural, urban), and country of residence (Benin, Cameroon, Gambia, Liberia, Madagascar, Mali, Nigeria, Rwanda, Sierra Leone, Zambia) were included as confounding variables for the study (see Table 1).

**Table 1:** Survey years, sample and number of women who sought help for intimate partner violence across countries.

Country	Survey year	Weighted sample	Number of women who sought help
Benin	2018	1,364	480
Cameroon	2018	2,105	808
Gambia	2020	960	260
Liberia	2020	1,311	594
Madagascar	2021	2,204	691
Mali	2018	1,580	303
Nigeria	2018	2,312	780
Rwanda	2020	967	479
Sierra Leone	2019	2,560	1,132
Zambia	2018	3,254	1,202
**Total**		**18,617**	**6,728**

### Analytical procedure

Descriptive and inferential analyses were carried out. The descriptive analysis looked at the bivariate analysis between the country and outcome variables. It also showed the frequency and proportions of the background characteristics by the outcome variables. Using a binary logistic regression model, a multivariate analysis was utilized to determine the association between the outcome variables and the explanatory variables. A binary logistic regression model was used based on the dichotomous nature of the outcome variable. Two models were fitted. The first model examined the relationship between the main independent variables (experienced emotional violence, experienced physical violence, experienced sexual violence and experienced childhood violence,) and the outcome variable (help-seeking behaviour). The second model examined the relationship between the outcome variable (help-seeking behaviour) and the main explanatory variables (experienced emotional violence, experienced physical violence, experienced sexual violence and experienced childhood violence) after adjusting for the confounding variables (place of residence, level of education, marital status, wealth status, women’s age, occupation, justifying wife beaten, partner domineering attitude and country variable). Each variable was subjected to a multicollinearity test, which revealed a mean-variance inflation factor (VIF) of 2.62 for the variables in the models. According to Chatterjee et al. [22], a VIF score higher than 10 indicates the presence of multicollinearity. Adjusted odds ratios with 95% confidence intervals were calculated for each variable. Stata was used to analyse the data (version 17). The results were sample-weighted to account for any under- or over-sampling in the sample.

## Results

### Descriptive statistics

**Fig 1:**
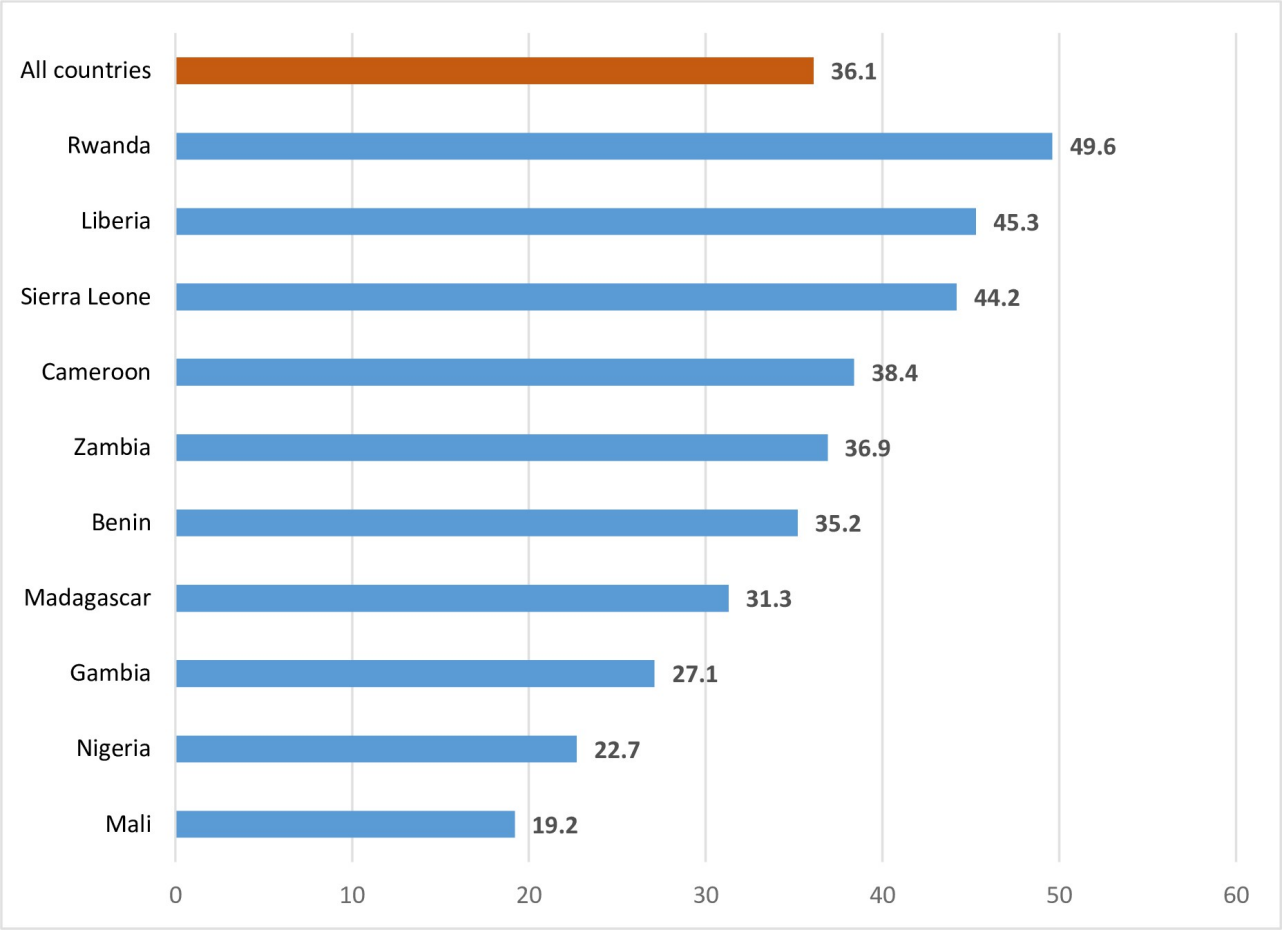
A graph on the percentages of women who sought help for intimate partner violence across countries.

The proportion of women who sought help for intimate partner violence was 36.1 percent (see Fig 1). This ranged from 19.2 percent in Mali to 49.6 percent in Rwanda (see Table 1).

Table 1 presents the ten countries included in the study and the years in which the surveys (2018–2021) were conducted. It also includes the weighted sample contribution of each country with the respective number of women who sought help for intimate partner violence.

Women who experienced emotional violence (44.2%), physical violence (40.5%), sexual violence (40.3%), aged 35–39 years old (38.2%), from rural areas (36.4%), with primary education (40.7%), with poorest wealth status (37.2%), married (33.5%), separated (46.7%), who justify wife beaten (36.1%), who had partner domineering attitude (38.5%) had the highest proportion of seeking for help for intimate partner violence (see Table 2).

**Table 2:** Background characteristics women who sought help for IPV.

Background Characteristics	Frequency	Proportion of women who sought help	X^2^ (p-value)
**Type of Violence**			
Experienced emotional violence			X^2^ = 704.6 (<0.001)
No	7,958	25.3	
Yes	10,659	44.2	
Experienced physical violence			X^2^ = 391.2 (<0.001)
No	5,620	26.2	
Yes	12,997	40.5	
Experienced sexual violence			X^2^ = 38.1 (<0.001)
No	14,391	34.9	
Yes	4,226	40.3	
Experienced childhood violence			X^2^ = 102.9 (<0.001)
No	15,094	37.6	
Yes	3,523	30.1	
**Demographic variables**			X^2^ = 18.5 (0.005)
*Age*			
15–19	924	32.5	
20–24	2,783	35.3	
25–29	3,870	35.4	
30–34	3,572	37.0	
35–39	3,265	38.2	
40–44	2,320	36.0	
45–49	1,883	35.9	
*Residence*			X^2^ = 0.2 (0.642)
Urban	7,404	35.7	
Rural	11,213	36.4	
*Level of education*			X^2^ = 33.2 (<0.001)
No education	6,257	33.7	
Primary	6,000	40.7	
Secondary	5,576	35.4	
Higher	783	33.9	
*Wealth status*			X^2^ = 23.5 (<0.001)
Poorest	3,352	37.2	
Middle	3,792	35.5	
Richer	3,908	36.6	
Richest	3,703	34.7	
*Marital status*			X^2^ = 248.5 (<0.001)
Married	13,571	33.5	
Cohabiting	2,360	41.9	
Widowed	570	36.9	
Divorced	787	46.6	
Separated	1,329	46.7	
*Occupation*			X^2^ = 76.0 (<0.001)
Not working	3,523	30.6	
Working	15,094	37.4	
**Sociocultural variables**			
*Justifying wife beaten*			
No	9,278	36.1	X^2^ = 2.30 (0.130)
Yes	9,339	36.1	
*Partner domineering attitude*			
No	3,701	26.6	
Yes	14,916	38.5	

### Inferential statistics

#### Binary logistic regression

The study found experienced emotional violence, physical violence, childhood violence, level of education, wealth status, marital status, occupation had significant associations with help-seeking behaviour of victims of intimate partner violence.

Women who experienced violence in their childhood (aOR = 0.75, CI = 0.69, 0.82) have a lower likelihood of seeking help compared to those who did not experience violence in their childhood (see Table 3). Women who had experienced emotional violence (aOR = 1.94, CI = 1.80, 2.08), and physical violence (aOR = 1.37, CI = 1.26, 1.48) have a higher likelihood of seeking help compared to those who have not. Women in secondary educational levels (aOR = 1.13, CI = = 1.02, 1.24) have a higher likelihood of seeking help compared to those with no education. Cohabiting women have a higher likelihood (aOR = 1.22, CI = 1.10, 1.35) of seeking help compared to married women (see Table 3).

**Table 3:** Binary logistic regression.

Background Characteristics	Model 1	Model 2
**Type of Violence**		
Experienced emotional violence		
No	Ref	Ref
Yes	1.93***(1.80, 2.07)	1.94***(1.80, 2.08)
Experienced physical violence		
No	Ref	Ref
Yes	1.44***(1.39, 1.55)	1.37***(1.26, 1.48)
Experienced sexual violence		
No	Ref	Ref
Yes	1.01(0.94, 1.09)	1.04 (0.97, 1.13)
Experienced childhood violence		
No	Ref	Ref
Yes	0.80***(0.74, 0.86)	0.75***(0.69, 0.82)
**Demographic variables**		
*Age*		
15–19		Ref
20–24		0.95(0.80, 1.12)
25–29		0.99(0.84, 1.16)
30–34		1.00(0.85, 1.19)
35–39		1.02(0.86, 1.20)
40–44		0.94(0.79, 1.12)
45–49		0.95(0.79, 1.14)
*Residence*		
Urban		Ref
Rural		1.01 (0.93, 1.10)
*Level of education*		
No education		Ref
Primary		1.16 **(1.07, 1.27)
Secondary		1.13**(1.02, 1.24)
Higher		1.10(0.91, 1.33)
*Wealth status*		
Poorest		Ref
Poorer		0.89** (0.81, 0.98)
Middle		1.01 (0.92, 1.12)
Richer		1.00(0.89, 1.13)
Richest		0.94(0.82, 1.08)
*Marital status*		
Married		Ref
Cohabiting		1.22***(1.10, 1.35)
Widowed		1.09(0.90, 1.33)
Divorced		1.65***(1.41, 1.93)
Separated		1.50***(1.32, 1.70)
*Occupational status*		
Not working		Ref
Working		1.33***(1.22, 1.46)
**Sociocultural variables**		
*Justifying wife beaten*		
No		Ref
Yes		0.94 (0.88, 1.01)
*Partner domineering attitude*		
No		Ref
Yes		1.29***(1.19, 1.41)
**Country**		
Benin		Ref
Cameroon		1.00 (0.85, 1.17)
Gambia		0.86 (0.71, 1.05)
Liberia		1.74***(1.47, 2.05)
Madagascar		0.71***(0.61, 0.83)
Mali		0.41***(0.34, 0.49)
Nigeria		0.94(0.80, 1.09)
Rwanda		1.47***(1.23, 1.76)
Sierra Leone		1.54***(1.33, 1.78)
Zambia		1.03(0.89, 1.20)

Ref = reference category

**p<0.01

***p<0.001 OR = Odds Ratio

CI = Confidence interval

Also, working women have a higher likelihood (aOR = 1.33, CI = 1.22, 1.46) of seeking help compared to women who are not working. Women whose partners have domineering attitudes have a higher likelihood (aOR = 1.29, CI = 1.19, 1.41) of seeking help compared to women who do not have a domineering attitude. Women from Sierra Leone (aOR = 1.47, CI = 1.23, 1.76) and Rwanda (aOR = 1.47, CI = 1.23, 1.76) have a higher likelihood of seeking help compared to women from Benin (see Table 3).

## Discussion

This study serves as one of the first attempts to explore holistically the influence of type of violence on help-seeking behaviour of victims of intimate partner violence using nationally representative cross-sectional data from ten sub-Saharan African (SSA) countries by considering the interplay between women’s socio-demographic characteristics and sociocultural factors. Our findings demonstrate a low rate of IPV victims seeking help among the sampled population, especially in Mali (19.2%). Out of the 18,617 IPV victims only about one-third (36.1%) sought help. The low rate of IPV victims seeking help in this study corroborates other studies in SSA countries [13] and other developing countries [18] that also revealed low rates of IPV victims seeking help. The plausible explanation of these findings may be due to the influence of the type of violence IPV victims experienced, and the role of some socio-demographic and cultural factors that may limit victims’ desire to seek help. In our study sample, the factors that significantly influenced IPV victims’ likelihood to seek help or not were: experience of emotional and physical violence, experience of childhood violence, education, wealth, marital status, occupation, and partner domineering attitude.

In this present study, we observed that IPV victims who experienced emotional and physical violence were more likely to seek help compared to their counterparts who did not. These findings are consistent with other studies in Turkey [15], and Nigeria [13]. However, the experience of sexual violence did not significantly influence victims’ help-seeking behaviour. In the cultural context of most SSA countries, issues of sexual acts are held in secrecy and its discussion in the open is deemed as an abomination. Hence, victims of sexual violence may be unlikely to seek help due to the fear of shame and stigmatization [23]. Another probable reason for these results is that, in the context of intimate relations, sexual activities are usually viewed as a prerogative responsibility, and issues of sexual violence in such relations may be taken lightly or normalized.

Another important observation was that IPV victims ever experienced childhood violence were less likely to seek help. Similar findings have been found in another study [21], which revealed that IPV victims ever experienced their father beating their mother during childhood were less likely to seek help. The possible reason for this result could be that IPV victims who experienced childhood violence might have normalized or internalized some violating acts blurring their definition of some forms of intimate partner violence as a problem and a need to disclose it or seek help. Based on this finding, it is imperative for earlier identification of IPV and intervention to demystify this culture of IPV normalization to enhance women’s zeal to seek help. However, this result contrasts the findings of Rowan, Mumford and Clark [24] who observed in India that IPV victims who observed their father abuse their mother were more likely to seek help from formal institutions.

The educational status of women showed a significant positive influence on the likelihood of IPV victims seeking help. We observed that the likelihood of IPV victims seeking help rises with an increase in their level of education. This finding corroborates several studies conducted in Turkey [15], Bangladesh [25], and other developing countries [18]. This could be to the point that educated women are more likely to be aware of the legalities revolving around IPV and hence recognize that it is unacceptable. Also, educated women are more likely to be aware of existing structures and institutions where they could seek help.

Consistent with prior research, women who were working had higher odds of seeking help for IPV compared to their counterparts who were not working [18, 21]. Working women may possess the financial means to access services for intimate partner violence. Additionally, this study revealed that compared to married women IPV victims with past intimate relations (divorced and separated) and cohabiting were more inclined to seek help. This finding is aligned with previous studies [13, 26]. The possible explanation for this could be that as intimate relations grow to enter into marriage couples’ commitments and love increase and women may begin to normalize certain acts considered as IPV, hence are less likely to report and seek help for IPV.

This study also draws attention to the role of sociocultural factors in predicting the likelihood of IPV victims’ decision to seek help. Thus, our analysis captured husband’s domineering behaviour as an important factor that links to the sociocultural norms influencing help-seeking behaviour of IPV victims. We found that women with domineering husbands had higher odds of seeking help for IPV. This finding affirms previous research by Tenkorang and colleagues [13] in Nigeria. The plausible reason could be that women with domineering husbands might experience more severe forms of intimate partner violence, motivating them to seek help as a coping mechanism or as a response to heightened danger, hence influencing their higher odds of seeking help.

### Policy implications and further research

The findings of this study have important policy implications for interventions aimed at reducing intimate partner violence (IPV) and increasing help-seeking behaviour among IPV victims in sub-Saharan African (SSA) countries. The low rate of IPV victims seeking help in this study suggests that there is a need to increase awareness of the available services and resources for IPV victims, especially in Nigeria where the rate was particularly low.

The study also identified a range of factors that influence IPV victims’ decision to seek help or not, including the type of violence experienced, childhood violence, education, wealth, marital status, occupation, and partner’s domineering attitude. These factors should be considered when designing and implementing interventions aimed at increasing help-seeking behaviour among IPV victims.

Another policy implication is to increase access to education, particularly for women, as the study found that educational status is positively associated with the likelihood of IPV victims seeking help. Governments in SSA countries should prioritize policies and programs that promote and ensure access to quality education for girls and women, female gender empowerment, establish clear reporting lines, and abolish certain socio-cultural practises, as these have the potential to reduce IPV and increase help-seeking behaviour.

Also, there is a need to develop and implement culturally appropriate interventions that challenge the normalization of IPV, particularly among those who have experienced childhood violence. This can be achieved through community awareness campaigns and interventions that challenge traditional gender roles and norms and promote positive and healthy relationships.

Given the influence of partner domineering behaviour on IPV victims’ likelihood of seeking help, interventions should focus on engaging men in efforts to prevent IPV and promote gender equality. This could be achieved through community-based programs that promote healthy and respectful relationships, and involve men as allies in the prevention of IPV. Based on this, further study is critical to explore the effectiveness of community resources, support networks, and grassroots initiatives in promoting help-seeking behavior and providing a safe environment for victims.

Also, further longitudinal studies are recommended to track the trajectories of IPV victims over time. This could help in understanding the changes in help-seeking behavior, especially about experiences of childhood violence. This type of study can provide insights into the dynamics of IPV and the factors influencing victims’ decisions to seek help at different stages of their lives.

Finally, it is encouraging for further studies to explore in more detail how cultural norms, attitudes, and beliefs contribute to the low rates of seeking help, especially in the context of sexual violence. Understanding these cultural nuances can inform targeted interventions to reduce stigma and encourage help-seeking.

### Strengths and limitations of the study

This study connotes several strengths: it draws on nationally representative cross-sectional data from ten sub-Saharan African countries, providing a robust sample for the analysis. Secondly, it takes a holistic approach to explore the influence of types of violence on help-seeking behaviour of victims of intimate partner violence, considering the interplay between women’s socio-demographic characteristics and sociocultural factors. This provides a nuanced understanding of the factors that influence help-seeking behaviour. Overall, this study provides important insights into the factors that influence the help-seeking behaviour of victims of intimate partner violence in sub-Saharan. However, there are several limitations to consider when interpreting the findings: This study used cross-sectional data, which limits the ability to establish causal relationships between variables. Longitudinal data would be necessary to explore the temporal relationship between the factors examined in this study and the help-seeking behaviour of IPV victims. Also, the data used in this study relies on self-reported experiences of intimate partner violence and help-seeking behaviour, which may be subject to social desirability bias or recall bias. Participants may underreport or overreport their experiences of violence or their help-seeking behaviour due to shame, fear, or memory distortion. Other critical dimensions of IPV such as spiritual and financial abuses were not considered in this due to the unavailability of data in the DHS dataset. Recognizing the full spectrum of abuse, including spiritual and financial aspects, is crucial for comprehensive understanding and effective intervention in cases of IPV. It underscores the importance of addressing various forms of power and control dynamics within intimate relationships to ensure the safety and well-being of victims. Lastly, this study was conducted in only 10 sub-Saharan African countries, and the findings may not be generalizable to other regions or cultural contexts. The cultural and social norms around gender and intimate partner violence can vary significantly across different regions and countries.

## Conclusion

This study sheds light on the low rate of IPV victims seeking help in sub-Saharan African countries, particularly in Nigeria. The study findings revealed that the type of violence experienced, socio-demographic factors such as education, wealth, marital status, and occupation, as well as sociocultural factors such as childhood violence and partner domineering attitude, significantly influence IPV victims’ likelihood to seek help. The study highlights the importance of early identification of IPV and intervention to demystify the culture of IPV normalization to enhance women’s willingness to seek help. The study’s findings suggest that education is crucial for increasing women’s awareness of the legalities surrounding IPV and available structures and institutions for seeking help. Additionally, working women and those with past intimate relations are more likely to seek help for IPV. Finally, the study’s analysis of the husband’s domineering behaviour emphasizes the role of sociocultural norms in predicting the likelihood of IPV victims seeking help. The study’s findings have significant implications for policy and practice in sub-Saharan African countries.

## Supporting information

S1 Checklist *PLOS ONE* clinical studies checklist. (DOCX)

S2 Checklist STROBE statement—checklist of items that should be included in reports of observational studies. (DOCX)
