# Supplementary material for: Influence of type of violence on women’s help-seeking behaviour: Evidence from 10 countries in sub-Saharan Africa
Source: PLoS One. 2024 Mar 8;19(3):e0297308. doi: 10.1371/journal.pone.0297308 (PMC10923450; doi:10.1371/journal.pone.0297308)
Supplement: S1 Checklist — (DOCX) [file pone.0297308.s001.docx]

***PLOS ONE* Clinical Studies Checklist**

***PLOS ONE* manuscript number: PONE-D-23-14651**

| **Complete the following if your study involved human participants or human subjects’ data. These questions should be addressed for prospective and retrospective studies.** | | |
| --- | --- | --- |
| 1. | Did you obtain ethics approval for this study?   - If yes, please upload (file type “Other”) the original approval document you received from your ethics committee. If the original document is in another language, please also provide an English translation.   ___ Uploaded _χ__ **N/A**   - If you did not obtain ethical approval, please explain why this was not required.  \| Since the study was a secondary data analysis of publically available survey data from the MEASURE DHS program, ethical approval, and participant consent were not necessary for this particular study. We requested DHS Program, and permission was granted to download and use the data for this study from ***http://www.dhsprogram.com***. There are no names of individuals or household addresses in the data files. \| \| --- \| |  |
| 2. | If your study involved human participants, please report in the Methods section when participants were recruited to the study.  ___ Completed ___**χ N/A** |  |
| 3. | If you are reporting a study of medical records or archived samples, please report in the Methods section the date range in which human subjects’ data/samples were collected and the date(s) when you conducted this study.  ___ Completed ___**χ** N/A |  |
| 4. | Please specify in the Methods section whether authors had access to information that could identify individual participants during or after data collection.  ___ Completed __**_χ** N/A |  |
| 5. | If you are reporting an observational study – i.e. cohort, case-control, and cross-sectional studies – we recommend that the work is reported as per the requirements of the STROBE guidelines, and that you provide a completed STROBE checklist as a Supporting Information file with your submission.  The STROBE checklist was developed to improve the reporting of observational human subjects research, and is available here: <http://strobe-statement.org/fileadmin/Strobe/uploads/checklists/STROBE_checklist_v4_combined_PlosMedicine.docx>.  __**_χ** **Completed**  ___ N/A |  |
| 6. | Please ensure that the author list and Corresponding Author entered in Editorial Manager match the author list and Corresponding Author in your manuscript file.  ___ **χCompleted** |  |
